# Questionnaires for the Assessment of Cognitive Function Secondary to Intake Interviews in In-Hospital Work and Development and Evaluation of a Classification Model Using Acoustic Features

**DOI:** 10.3390/s23115346

**Published:** 2023-06-05

**Authors:** Toshiharu Igarashi, Yumi Umeda-Kameyama, Taro Kojima, Masahiro Akishita, Misato Nihei

**Affiliations:** 1 Department of Human and Engineered Environmental Studies, The University of Tokyo, Kashiwanoha 5-1-5, Kashiwa 277-8563, Japan; 2 Graduate School of Medicine and Faculty of Medicine, The University of Tokyo, 3-1, 7-3-1 Hongo, Bunkyo-ku, Tokyo 113-0033, Japan; 3 Institute of Gerontology, The University of Tokyo, 3-1, 7-3-1 Hongo, Bunkyo-ku, Tokyo 113-8654, Japan

**Keywords:** gerontology, intake interview, cognitive function, audio processing, MFCC, mel-spectrum

## Abstract

The number of people with dementia is increasing each year, and early detection allows for early intervention and treatment. Since conventional screening methods are time-consuming and expensive, a simple and inexpensive screening is expected. We created a standardized intake questionnaire with thirty questions in five categories and used machine learning to categorize older adults with moderate and mild dementia and mild cognitive impairment, based on speech patterns. To evaluate the feasibility of the developed interview items and the accuracy of the classification model based on acoustic features, 29 participants (7 males and 22 females) aged 72 to 91 years were recruited with the approval of the University of Tokyo Hospital. The MMSE results showed that 12 participants had moderate dementia with MMSE scores of 20 or less, 8 participants had mild dementia with MMSE scores between 21 and 23, and 9 participants had MCI with MMSE scores between 24 and 27. As a result, Mel-spectrogram generally outperformed MFCC in terms of accuracy, precision, recall, and F1-score in all classification tasks. The multi-classification using Mel-spectrogram achieved the highest accuracy of 0.932, while the binary classification of moderate dementia and MCI group using MFCC achieved the lowest accuracy of 0.502. The FDR was generally low for all classification tasks, indicating a low rate of false positives. However, the FNR was relatively high in some cases, indicating a higher rate of false negatives.

## 1. Introduction

Dementia is “a chronic decline or loss of various cognitive functions, resulting in the inability to lead a normal daily or social life”, and is an acquired intelligence disorder [1,2]. Cognitive functions are essential for planning and carrying out daily activities such as cleaning, washing clothes, eating, and going out [3].

According to a Japanese government report, the number of elderly people with dementia is estimated to increase from 4.62 million in 2012 to 7.3 million by 2025 [4]. Approximately 10% of the total population will develop the disorder at some point in their lives [5], typically as a result of aging [6]; about 3% of those aged 65–74, 19% of those aged 75–84, and almost half of those aged 85 years and older have dementia [7]. This number is expected to nearly double every 20 years and increase to 152 million by 2050 [8]. The current medical science has not yet found a cure for dementia. However, drugs that slow the progression of dementia exist, and non-pharmacological therapies have shown some efficacy [9,10]. In addition, cognitive decline often begins with mild cognitive impairment (MCI), which has increased in recent years [11]. The most important measures against dementia are to detect the trend of cognitive decline as early as possible and intervene at an early stage.

Prevention, early detection, and early intervention for dementia are known to reduce medical costs. However, the lifestyles of the elderly are changing in Japan, and while it used to be common for two or three households to live together, the number of single people and elderly couples has been increasing in recent years [11]. As a result, it has become more difficult for child-headed households to notice the decline in the cognitive function of their parents and link this to support. In addition, the current comprehensive community care system does not provide sufficient outreach support because of the limitations of the system and human resources [12,13].

Rigorous dementia testing methods include positron emission tomography (PET) and magnetic resonance imaging (MRI), but these are time-consuming and require the use of expensive testing equipment [14]. Other methods, such as the Mini-Mental State Examination (MMSE) and the Revised Hasegawa Dementia Scale (HDS-R), are quick and easy screening methods [15,16]; however, because the questions on the test form are fixed, they can be memorized by the examinee and are unsuitable for periodic monitoring. Therefore, screening for dementia based on the verbal abilities of the elderly has attracted attention in recent years.

## 2. Related Works

### 2.1. Screening for Dementia and Its Challenges

Screening tests are more commonly used in medical practice, especially the Revised HDS-R and the MMSE. These tests have shown high reliability, validity, and screening accuracy as indicators of cognitive function in the elderly, and are used in a variety of settings, especially in medical institutions. However, most of these cognitive function tests are conducted in a format that questions ability through tests with fixed correct and incorrect answers. Therefore, many elderly people are reluctant to take cognitive function tests because of the psychological burden and the potential stigma associated with dementia [17]. For example, 70% of patients with Alzheimer’s disease (AD) and 47% of those without dementia have been reported to experience psychological distress during cognitive function tests, and 16% of AD patients show anxiety or anger during tests [18]. Such distress and anxiety caused by cognitive function tests in the early stages of treatment may affect the subsequent therapeutic relationship, and the relationship between the healthcare provider and the patient may also affect the effectiveness of treatment [19]. Because of this influence on the therapeutic relationship, there is sometimes resistance on the part of healthcare providers to conduct cognitive function testing [20].

Therefore, in recent years, screening for dementia based on the verbal abilities of the elderly has attracted attention. Such screening is not physically invasive and does not require long-term restraint at medical institutions. In addition, it is attracting attention because cognitive function can be monitored on a regular basis and it may be possible to detect changes in cognitive function over time.

### 2.2. Spontaneous Conversation Test

In general, patients with dementia are known to have reduced language skills compared with healthy controls. It has been shown that people with dementia have conversational characteristics such as an inability to recall accurate information, delayed responses, unclear content, and reluctance to answer more than what is asked, and it has been reported that collecting these characteristics can provide useful information for diagnosis [21]. Many studies have been conducted screening for dementia based on language ability.

A study by Renato et al. investigated the effectiveness of screening for dementia using a word list learning test in Japanese subjects [22]. Characteristics included percent correct, recall, accuracy, F1 value, and AUC, with a sample size of 50 for the healthy group and 50 for the dementia group. The highest accuracy was from a word list learning test, at 75%. Oveisgharan et al.’s study showed that performance on a language processing task could be used to help diagnose MCI [23]. The subjects were English speakers, and the features were accuracy in vocabulary testing, category fluency, grammar, and speech processing. The sample size was 80 healthy subjects and 40 MCI subjects. The best feature for classifying MCI and healthy subjects was accuracy in speech processing with an AUC of 0.82.

In recent years, there has been a gradual increase in the number of studies [24] to discriminate patients with dementia from healthy subjects using machine learning. According to previous systematic reviews [25], machine learning-based assessments of cognitive function can be classified into four main categories, depending on the type of features employed: (i) linguistic features, (ii) acoustic features, and (iii) other types of features such as facial expressions or features that depict specific shapes. Acoustic features can be extracted using three types of features: (1) analysis of prosodic features, (2) voice quality features, and (3) articulatory features.

Prosody refers to the rhythm and melody of one’s speech [26]. Examples of computable temporal prosodic features from recorded speech signals include the duration of voiced segments, duration of silent segments, loudness, measures of periodicity, fundamental frequency (F0), and many other similar features [27]. The study by König et al. evaluated an automatic detection method using speech features such as mean F0, pitch variability, mean intensity, intensity variability, speech rate, and pauses in 32 patients with dementia and 32 healthy subjects [28]. The results showed that the classification accuracy was 0.5 for the dementia patients and 0.78 for the healthy subjects. In a study by Eduardo et al., the experiment involved twenty participants, half of whom had been diagnosed with mild dementia, and the other half were healthy controls. The automatic prosodic analysis was conducted on a reading task to measure the prosodic attributes in both the mildly demented patients and the healthy elderly controls. Their approach extracted twelve prosodic features from the speech samples. An impressive classification accuracy of 85% was achieved using just four of these prosodic features [29].

Voice quality features are a method of estimating cognitive decline using the quality of conversational voice generated by the elderly. In this context, jitter (variation in glottal pulse timing), shimmer (variation in glottal pulse amplitude), and harmonic-to-noise ratio (ratio of formant harmonics to anharmonic spectral peaks) are used to measure voice quality during voiced sounds. According to J. C. Hailstone et al., airflow through the lungs and glottis is said to be altered by cognitive decline. This feature can also be measured from the speech signal using several separation algorithms [30]. A study by Cogollor et al. classified voice data from 27 dementia patients and 27 healthy subjects using five features: shimmer, jitter, HNR, GNE, and NHR. The classification model used logistic regression, and the classification accuracy was reported to be 0.75 [31]. Nakamura et al. used six features, i.e., voice break, pitch variability, shimmer, jitter, Energy, and MFCC, to classify voice data from 31 dementia patients and 31 healthy subjects. Data were classified [32]. A convolutional neural network (CNN) was used as the classification model, and a classification accuracy of 0.794 was reported. Lin et al. used five features, namely HNR, NHR, shimmer, jitter, and formant frequencies, to classify voice data from 34 dementia patients and 34 healthy subjects. The classification model was based on Support Vector Machine (SVM), and the classification accuracy was reported to be 0.82 [33].

Articulation features are methods that focus on the connections between sounds. Several spectral features that capture sound connections have been used in the clinical speech literature to measure the acoustic manifestations of cognitive language disorders. The spectral centroid is a measure used in digital signal processing that characterizes the spectrum and indicates where the center of gravity of the spectrum lies. It can be calculated for each frame of the analyzed speech signal [34]. Others include the calculation of statistics related to additional formant harmonic frequencies (i.e., F1, F2, F3) [35] or the calculation of vowel space area [36]. Because of the non-stationarity of speech signals, time-frequency signal processing algorithms can also be used. For example, Mel Frequency Cepstral Coefficients (MFCC) may also be used by applying a Mel scale filter bank [37]. Ghosh et al. performed an emotion recognition task using MFCC. Two speech datasets, TESS and RAVDESS, were used, and classification was performed using CNN [38]. The accuracy was 0.846, indicating that speech analysis using MFCC is effective. Another study by Zhu et al. performed a cough speech recognition task using a Mel-spectrogram. The dataset used was speech data of coughs collected in-house and classified by CNN [39]. The accuracy was 0.83, indicating that speech analysis using Mel-spectrograms is effective.

Thus, MFCC and Mel-spectrograms are widely used in cognitive function classification tasks; for MFCC, the method proposed by Rabiner [40] et al. is one of the most commonly used features in speech recognition. This method is obtained by dividing the speech signal into frames, performing a Fourier transform on each frame, and then extracting features in the frequency domain. By using the logarithmic spectrum of frequencies, this method can extract features based on frequency sensitivity in the human auditory system. The Mel-spectrogram, on the other hand, is obtained by dividing the frequency spectrum into fixed frequency bands and calculating the amplitude for each band. This allows the frequency axis to be converted to a Mel scale and features based on the human auditory system to be extracted, and it is known that CNNs outperform other classification models in image classification tasks using MFCC and Mel spectrograms, as studied by Han et al. [41]. In cognitive function classification tasks, both are widely used; however, which one is more accurate depends on the task, and both need to be validated.

### 2.3. Dataset Task Challenges for the Test

The type of task a patient is asked to perform has a significant impact on its clinical applicability. The ADReSS Challenge dataset consists of audio recordings of participants’ descriptions of pictures [42]. Other datasets provided by Shihata et al. to discriminate between healthy elderly and MCI also included tasks that require participants to describe pictures and animations [43]. Such picture and animation description tasks are not considered natural conversations because the display must be available during the conversation [44].

The Conversational Assessment of Neurocognitive Dysfunction (CANDy) [45] Test by Oba et al. is based on daily conversation and asks 15 questions, including “repeating the same questions during conversation”, “talking in a roundabout way”, and “not knowing the current time, date, season, etc.”. The total score is 30 points, with higher scores indicating greater cognitive decline. The sensitivity and specificity for AD were 86.2% and 94.5%, respectively, indicating high screening accuracy. However, the CANDy provides few instructions regarding the content of the conversation, making it difficult to make comparisons between the participants. Furthermore, the overall evaluation of conversations is based on a qualitative assessment by the examiner, which raises questions regarding reproducibility.

In psychiatry and geriatrics, intake interviews are conducted with patients to elicit relevant information to provide comprehensive support in treatment. Intake interviews are the most common type of interview in clinical psychology, occurring when a client first comes to a clinician for help. Interviews are often conversational in nature and considered beneficial to both parties, as the inclusion of personal conversation topics can lead to a mutual understanding of the communication styles of the interviewer and patient. In practice, interviews are conducted by nurses, psychologists, and social workers about the patient’s life history and current living situation, and this information is shared with physicians and medical teams for smooth treatment and discharge planning [19]. In some cases, the examiner’s findings on the patient’s cognitive function are also included, but these are findings based on experience and are often difficult for nurses and psychologists who have just been assigned to the patient’s care [20].

### 2.4. Motivation

It would reduce the burden on hospital staff and patients if cognitive function could be estimated mechanically from conversations conducted with patients in practice. However, when considering their constant use in hospitals, it is necessary to develop question items that can be used universally, to some extent, for any patient. Therefore, the purpose of this study was to develop, together with psychologists’ original interviews, items for intake interviews that can be used in hospital practice.

Additionally, we verified the accuracy of the classification of data collected from the interviews. In a previous study, classification of the healthy elderly group and the AD group was conducted; however, it is not yet known whether the same level of accuracy can be achieved when cognitive decline has progressed beyond MCI. Therefore, we also developed and validated the accuracy of a classification model based on acoustic features that can perform multilevel classification of three types of dementia: moderate dementia, mild dementia, and MCI groups. The MFCC and Mel-spectrogram are used as acoustic features, and the difference in accuracy is also compared.

## 3. Methods

### 3.1. Methods for Creating Life History Interview Items for Intake Interviews

Life history interview items must contain content that can be used in actual hospital practice. Therefore, the interview items were developed according to the following protocol:

1. The author will attend an intake interview with a psychologist at the University of Tokyo Hospital and survey the questionnaire items.
2. Delete items deemed unimportant or duplicated from the questionnaire items and develop a preliminary draft.
3. Five licensed psychologists working at the University of Tokyo Hospital checked the draft, made additions and revisions, and changed the order of questions.
4. After confirmation by the authors and supervisors, a final version was prepared.

### 3.2. Questionnaire

The questionnaire consisted of 30 questions in 5 categories. The categories were (1) process before coming to the hospital, (2) life history, (3) ordinary life, (4) interests and concerns, and (5) plans for the rest of the day, with questions related to each category listed below (Table 1).

### 3.3. Questioner Reactions and Additional Questions

To ensure that the conversation did not end with a short response when asking a question, the questioner implemented two types of reactions: one was to repeat the same information as the participant, mirroring them to encourage the participant to continue talking after the participant’s response. The second was a reaffirmation of the participant’s response of “nothing in particular”, followed by the question, “If you had to give a strong answer, what would it be?” The second was a reconfirmation of the question, “If you could force me to do something, what would it be?”

## 4. Evaluation

### 4.1. Experimental Environment

**Figure 1:**
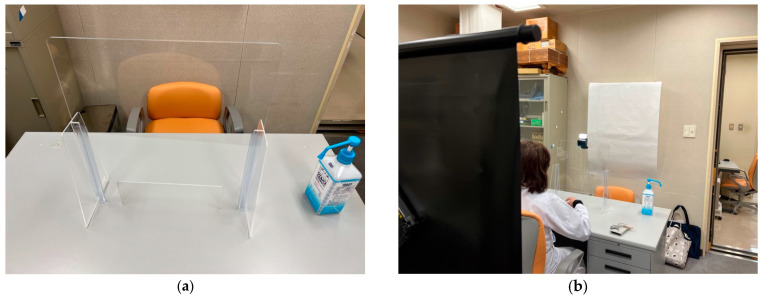
(**a**) A table partitioned by an acrylic board. (**b**) The interviewer sat on the far side and the participant sat on the front side.

The experiment was conducted in the examination room at the University of Tokyo Hospital (Figure 1). The participants and questioners sat face-to-face at a desk in the examination room and asked questions. Audio- and time-lapse images were recorded using a recorder and a small camera (GoPro Hero 10) [46]. To prevent infection, the questioner wore a face guard and the participants’ hands were disinfected when they entered the room. The room was ventilated with a circulator, and an acrylic board was used as a partition between the participant and interviewer. Furthermore, the desks and chairs were disinfected with alcohol spray and paper napkins after the participants left the room.

### 4.2. Participants

Participants were recruited from August to September 2022. Inclusion criteria were patients aged 65 years or older who had been diagnosed with dementia by a physician. Those who were able to give their consent were invited to participate after an explanation of the research outline was given. The consent form was obtained from the patient only if the patient came to the hospital or from a relative if the patient was accompanied by a relative. This study was approved by the ethics review committee of the University of Tokyo Hospital.

### 4.3. Screening Tests

The tests were divided into (a) the MMSE, (b) the Geriatric Depression Scale (GDS), and (c) a life history interview during the intake interview. The MMSE is one of the most common screening methods for detecting dementia. It is a 30-point cognitive function test consisting of 11 items: time and place perception, immediate and delayed wordplay, calculation, object calling, and sentence recitation, three-step verbal command, written command, and graphic imitation. In the MMSE, a score of 23 points or less indicates potential dementia (sensitivity of 81%; specificity of 89%), and a score of 27 points or less indicates potential MCI (sensitivity of 45–60% and specificity of 65–90%) [47,48,49,50].

It has been reported that the results of cognitive function screening tests show no significant changes within three months. Therefore, if the patient had undergone a cognitive function test at the same hospital within 3 months, the test was omitted, and the most recent test result was used to reduce patient burden. The GDS is a screening test used to assess depression in older adults. As it is known that the amount of conversation is reduced when a person is depressed, a shortened version of the GDS-15 [51] was administered to ascertain participants who were above the cut-off value [52].

### 4.4. Machine Learning of the Obtained Audio Data

As a preprocessing step, the recorded speech data were manually annotated with 4827 response data, in which the participants were speaking continuously without any additional questions by the questioner and clipped. If a participant was silent for more than one second, the second was counted. For the 4827 clipped response data, 3990 responses with utterances of 1 s or longer were included. Conversion to MFCC and Mel-spectrograms was performed using Librosa, an audio processing package [53]. MFCC was represented by time on the horizontal axis and hertz on the vertical axis, while the Mel-spectrogram was drawn on a decibel basis on the horizontal and vertical axes. Both were processed at a sampling frequency of 44,100 Hz.

**Figure 2:**
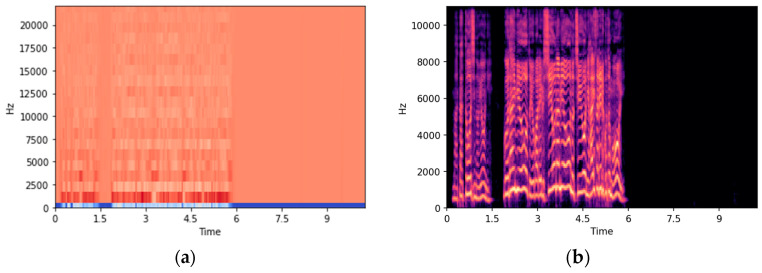
(**a**) Figure converted from a wav data file by MFCC. (**b**) Figure converted from a wav data file by Mel-spectrogram.

A convolutional neural network (CNN) was used as a classification model for images from which the features were extracted. It can automatically extract valid features and achieve high recognition accuracy in various tasks such as general object recognition [54]. Image data converted using Librosa is output at 72 dpi with an aspect ratio of 6.0 and 4.0 by default (6 × 72 = 432 pix in width and 4 × 72 = 288 pix in height). Since the image was too large as an input image, the input to the CNN was image data resized to 100 × 100 pixels from the MFCC and Mel-spectrogram images (Figure 2).

In this program, a CNN with two convolutional layers and two pooling layers was used to classify three classes of images. The optimization method was performed using the most commonly used optimizer, Adam. The parameter batch size of the model was selected from [2,4,8,16,32] using grid search. Grid search tries different parameter combinations and selects the best parameter that gives the highest cross-validation score. The number of epochs was set to 200. Early stopping was implemented to prevent overtraining. In other words, to prevent overtraining, training can be terminated early when specified conditions, such as the number of epochs or the value of the loss function, are reached.

To evaluate the performance of the model during training, 10-split cross-validation was performed. In this study, the dataset was divided into 10 pieces of data (9 for training and 1 for test), the model was trained 10 times, and a test set score was calculated for each training. The final score was computed using the mean and standard deviation of the 10 scores. Tenfold cross-validation is the generally recommended cross-validation method. While it is possible to use 5 or 8 folds, these folds are not recommended due to the smaller size of the training and test sets and the potential for inadequate evaluation of model performance. On the other hand, if 15- or 20-split splitting methods are used, the size of the training and test sets will be very small, which may lead to an overestimation of the model’s performance. Therefore, 10-split cross-validation is used in this paper.

### 4.5. Evaluation Indicators

Performance was evaluated in terms of accuracy, precision, reproducibility, F1-score, false discovery rate (FDR), and false negative rate (FNR). Since there is bias in the amount of data in each group in this sample, a weighted average was used to evaluate performance. Accuracy is a metric that shows the proportion of correct predictions out of all predictions made. Precision is a metric that shows the proportion of true positive cases among those that are predicted to be positive. Recall is a metric that shows the proportion of true positive cases that are correctly identified as positive by the model. F1-score is a metric that is the harmonic mean of precision and recall, which measures the accuracy of the model in predicting the positive class. False discovery rate (FDR) is a metric that shows the proportion of false positives among all positive predictions made. False negative rate (FNR) is a metric that shows the proportion of false negatives among all negative predictions made.

## 5. Results

In total, 29 people (7 men and 22 women) between the ages of 72 and 91 participated in the experiment. The MMSE results showed that 12 participants had moderate dementia with MMSE scores of 20 or less, 8 participants had mild dementia with MMSE scores between 21 and 23, and 9 participants had MCI with MMSE scores between 24 and 27. In addition, 27 patients scored below 7 in GDS, the cut-off value for the first state, and 2 patients scored higher than the cut-off value. However, given that the diagnosis of depression was not made during the specialist’s examination immediately after the test, we did not exclude this person from the study.

From the 12 participants in the moderate dementia group, 1542 response data were obtained. The average number of seconds of silence per response was 2.776. From the eight members of the mild dementia group, 1390 responses were obtained. The average duration of silence per response was 1.849. A total of 1895 responses were obtained from nine participants in the MCI group. The average number of seconds of silence per response was 1.589 s.

Since response data of less than 1 s were unclassifiable, these data were excluded. We used 1432 responses from the moderate dementia group, 1151 responses from the mild dementia group, and 1406 responses from the MCI group for classification. Multi-classification was performed with 3989 data from all groups combined. Binary classification between the moderate and the mild dementia group was performed with 2583 data, binary classification between the moderate dementia group and the MCI group was performed with 2838 data, and binary classification between the mild dementia group and the MCI group was performed with 2557 data.

**Figure 3:**
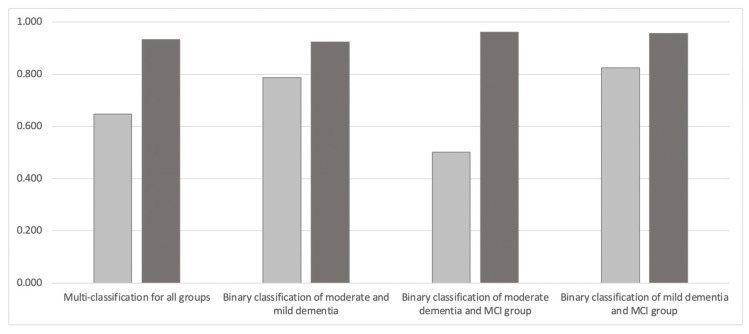
Comparison of classification accuracy between MFCC and Mel-spectrogram. Light gray represents MFCC and dark gray represents Mel-spectrogram.

Figure 3 and Table 2 show the results of using MFCC and Mel-spectrogram for both multi-classification and binary classification. With multi-classification (n = 3989), the accuracy of the model using MFCC was 0.647 and that using Mel-spectrogram was 0.932, with Mel-spectrogram achieving higher accuracy than MFCC.

This tendency was also the same for the binary classification model of the moderate and mild dementia group (n = 2583), which achieved 0.923 accuracy for the Mel-spectrogram compared to 0.787 for MFCC. The binary classification model of the moderate dementia and MCI groups (n = 2838) achieved 0.961 accuracy with the Mel-spectrogram compared to 0.502 with MFCC. The binary classification model of the mild dementia and MCI groups (n = 2557) achieved 0.957 accuracy with the Mel-spectrogram compared to 0.824 with the MFCC.

## 6. Analysis

### 6.1. Analysis of the Number of Seconds of Silence

**Figure 4:**
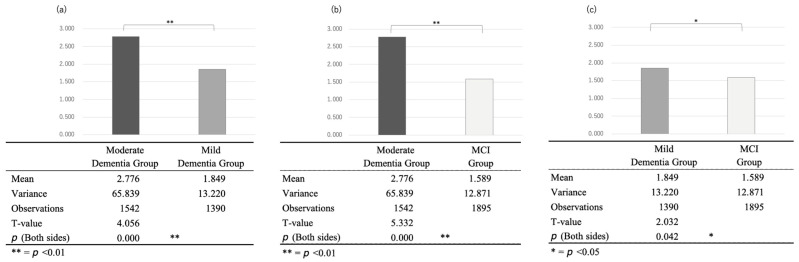
Comparison of the seconds of silence between the (**a**) moderate and mild dementia groups, (**b**) moderate dementia and the MCI groups, and (**c**) mild dementia and MCI groups.

Comparing the average number of seconds of silence per response in the three groups, we found 2.776 s for the moderate dementia group (12 people, 1542 samples), 1.849 s for mild dementia (8 people, 1390 samples), and 1.589 s for the MCI group (9 people, 1895 samples). A test of the difference of means without correspondence for the differences between each group showed significant differences (*p* < 0.01 for moderate/mild and moderate/MCI and *p* < 0.05 for mild/MCI, with t-values of 4.056, 5.332, and 2.032, respectively) (Figure 4).

This indicates that the average number of seconds of silence per case in the three groups, compared with the semi-structured intake interview using the same questions, tended to increase as cognitive function declined. In fact, the most accurate model (0.961) in the binary classification using the Mel-spectrogram was the intermediate dementia group and the MCI group, which differed the most in the number of silent seconds. Regarding a previous study’s suggestion that conversations become more verbose as cognitive function declines, the increase in the number of silent seconds in a conversation may be related to the use of the intake-interview-based open queries used in this study.

### 6.2. Analysis of the Sex Difference

This sample included 7 men and 22 women. Of the 7 males, 1 was classified as having moderate dementia, 3 as having mild dementia, and 3 as having MCI; of the 22 females, 11 were classified as having moderate dementia, 5 as having mild dementia, and 6 as having MCI. Therefore, we compared the classification performance with male-only and female-only data to see if there were any gender differences in classification performance (Table 3).

Analysis of feature differences showed that Mel-spectrogram features generally outperformed MFCC features for both men and women in all task results; the accuracy increased from 0.882 (MFCC) to 0.936 (Mel-spectrogram) for men and from 0.725 (MFCC) to 0.905 (Mel-spectrogram) for women in the multi-valued classification task.

In general, classification accuracy tended to be higher for males, but it was higher for females than for males for the binary classification of mild dementia and MCI groups using the MFCC and for the binary classification of mild dementia and MCI groups using the Mel-spectrogram. In terms of differences in accuracy by gender, among all tasks, the highest accuracy was for the binary classification of moderate dementia and mild dementia, which was the same for both males (acc = 0.980) and females (acc = 0.962). As revealed in Section 6.1, there were significant differences in the number of silent seconds in moderate dementia, mild dementia, and MCI groups. In our sample, male patients with moderate dementia had the longest silent seconds in speech, which may affect the accuracy of the classification model reflecting the characteristics of cognitive decline.

## 7. Discussion

### 7.1. Classification Accuracy Using MFCC and Mel-Spectrogram

The accuracy of the classification model using the Mel-spectrogram was higher than that of MFCC for both the tri- and binary classification patterns. The highest classification accuracy was for the binary classification of mild MCI using the Mel-spectrogram (0.930), and the lowest accuracy was for the binary classification of moderate MCI using the MFCC (0.481). This may be because the Mel-spectrogram was better suited to show the features of the occurrence and percentage of silent seconds in the speech content, as shown in Figure 4. MFCC is obtained by converting the frequency spectrum into the Mel scale before applying a discrete cosine transform. On the other hand, the Mel-spectrogram is obtained by obtaining a spectrogram through the short-time Fourier transform before converting the frequency spectrum into the Mel scale. MFCC is an optimized feature to represent the shape of the frequency spectrum and is designed based on human auditory characteristics. Mel-spectrograms are also used to represent the frequency characteristics of audio signals, but they are considered to have more information than MFCC because they directly represent the distribution of energy.

Observing the images that failed classification in the test data, there was a tendency for the data to fail classification with characteristics that were different from the overall trend, such as fewer silent seconds in the speech of the moderate dementia group and fewer silent seconds in the MCI group. Even among patients with moderate dementia, not all utterances had long silent seconds, and conversely, even among MCI patients, not all utterances had short silent seconds. Although there were 30 questions in total, and all the response data were used in this classification, it is thought that some categories and individual questions significantly affect the accuracy of classification, while others, on the contrary, reduce it. Although the average interview with participants lasted about one hour, it is necessary to conduct interviews in a shorter time, especially in the context of hospital practice, to reduce the burden on hospital staff and patients. Therefore, a detailed analysis of each question item to create more refined questions will be a future task.

Another factor that contributed to achieving higher accuracy than classification using a dataset such as ADress was the larger dataset for training. In this study, thirty questions in five categories, rather than just one task, were asked, and the responses to each were obtained, which may have contributed to the high classification accuracy. Binary classification using Mel-spectrograms both exceeded 90% accuracy and may be useful as reference information when the MMSE or other tests cannot be performed. It may be possible to discriminate the speech of healthy elderly people from MCI and other targets; however, additional data are needed to determine the level of accuracy.

### 7.2. Future Work

In this study, a comparison of the classification accuracy of MFCC and Mel-spectrogram on a Japanese dataset was presented; however, whether reproducibility can be achieved for other languages requires further validation. However, Bonaventure et al. performed the emotion classification task on several speech datasets, including German, Italian, and British English, using image classification models, and found that the Mel-spectrogram-based model was quantitatively shown to be more accurate than the MFCC-based model [55]. Therefore, it is expected that the Mel-spectrogram will work better for German, Italian, British English, etc.

Furthermore, the input images in the image classification model were resized to 100 × 100 pixels. The appropriate size of input images in CNNs varies from task to task. Therefore, the input size should be adjusted based on the available GPU memory, and tools such as Oputuna have been developed to help calculate the optimal input size. Further verification is required to determine the resolution and image size that facilitate the capture of cognitive function features [56].

## 8. Limitations

Although this study also analyzed gender differences, the small amount of data is a limitation of this study. As noted above, there was only one male participant in moderate dementia, which should be re-examined with a larger sample size, as described in Section 7.2.

## 9. Conclusions

In this study, we developed interview items and a classification model using acoustic features to estimate cognitive function, assuming an intake interview with psychologists in a hospital. The interview items were prepared by the author, who witnessed the psychologist’s intake interview and deleted important or duplicated items from the questionnaire. The questionnaire consisted of thirty questions in five categories. The categories were as follows: (1) process before coming to the hospital, (2) life history, (3) ordinary life, (4) interests and concerns, and (5) plans for the rest of the day, with questions related to each category included in the tier below.

To evaluate the feasibility of the developed interview items and the accuracy of the classification model based on acoustic features, participants were recruited with the approval of the University of Tokyo Hospital, and 29 people (7 males and 22 females) aged 72 to 91 years participated. The MMSE results showed that 12 participants had moderate dementia with MMSE scores of 20 or less, 8 participants had mild dementia with MMSE scores between 21 and 23, and 9 participants had MCI with MMSE scores between 24 and 27. Mel-spectrogram generally outperformed MFCC in terms of accuracy, precision, recall, and F1-score in all classification tasks. The multi-classification using Mel-spectrogram achieved the highest accuracy of 0.932, while the binary classification of moderate dementia and MCI groups using MFCC achieved the lowest accuracy of 0.502. The FDR was generally low for all classification tasks, indicating a low rate of false positives. However, the FNR was relatively high in some cases, indicating a higher rate of false negatives.

Comparing the average number of seconds of silence per response in the three groups, we found 2.776 s for the moderate dementia group (12 people, 1542 samples), 1.849 s for mild dementia (8 people, 1390 samples), and 1.589 s for the MCI group (9 people, 1895 samples). A test of the difference of means without correspondence for the differences between each group showed significant differences (*p* < 0.01 for moderate/mild and moderate/MCI and *p* < 0.05 for mild/MCI, with t-values of 4.056, 5.332, and 2.032, respectively).

This sample included 7 men and 22 women. Therefore, we compared the classification performance with male-only and female-only data, respectively, to see if there were any gender differences in classification performance. From the results, Mel-spectrogram features generally outperformed MFCC features in all tasks for both men and women. The classification performance for men was higher than that for women in all tasks. Furthermore, binary classification of moderate and mild dementia was the easiest task for both men and women, with the highest accuracy and F1-score achieved by Mel-spectrogram features.

## Figures and Tables

**Table 1:** Intake interview questions.

**(1) Process before coming to the hospital** Q1. Where is your home? Q2. How long did it take you to get here today? Q3. After you left your home, how did you come here? Q4. What time did you leave home to come to the hospital today?
**(2) Life history** Q5. Where were you born? Q6. Do you have any siblings (if so, how many?) Q7. Which elementary school did you attend? Q8. What did you do after elementary school? (Which junior high school did you attend?) Q9. What did you do after graduating junior high school? (Which high school did you attend?). Q10. What do you do for work? (Do you have any memorable stories?) Q11. Are you married? (When was your wedding?) Q12. Do you have any children? (Where do your children live?)
**(3) Daily life** Q13. How do you usually spend your time? (Please tell us your approximate weekly schedule.) Q14. What time do you get up in the morning and go to bed? Q15. How often do you go out? (Where do you go most often?) Q16. Do you bathe every day? (Do you bathe in a bathtub?) Q17. How do you prepare your meals? (Do you eat three meals a day?)/What did you eat last night? Q18. How do you clean your house? (How often do you clean your house?) Q19. How do you do your laundry? (How often do you do it?)
**(4) Interests** Q20: What news have you been interested in on TV or the Internet recently? Q21: Please tell me about a sad event that happened to you recently. Q22: Please tell me about a recent unsettling event. Q23: Tell me about a recent event that made you angry. Q24: Tell me about a recent event that made you feel bad. Q25: Tell me about a recent event that surprised you. Q26: Tell me about a recent happy event that happened to you. When did it happen? Q27: Tell me about someone you admire. Q28: What are you passionate about these days?
**(5) Plans for the rest of the day** Q29: What are your plans for the rest of the day? (How will you get home?) Q30: When was the date of your last visit?

**Table 2:** The results of using MFCC and Mel-spectrogram for multi-classification and binary classification.

Feature Used	Classification Method	Accuracy	Precision	Recall	F1-Score	FDR	FNR	Number of Data
Mfcc	Multi-classification for all group	0.647	0.749	0.647	0.690	0.251	0.353	3989
	Binary classification of moderate and mild dementia	0.787	0.800	0.787	0.781	0.200	0.213	2583
	Binary classification of moderate dementia and MCI group	0.502	0.252	0.502	0.335	0.748	0.498	2838
	Binary classification of mild dementia and MCI group	0.824	0.838	0.824	0.819	0.162	0.177	2557
Mel-spectrogram	Multi-classification for all group	0.932	0.932	0.932	0.932	0.067	0.068	3989
	Binary classification of moderate and mild dementia	0.923	0.923	0.923	0.923	0.077	0.078	2583
	Binary classification of moderate dementia and MCI group	0.961	0.961	0.961	0.961	0.039	0.039	2838
	Binary classification of mild dementia and MCI group	0.957	0.957	0.957	0.957	0.043	0.043	2557

**Table 3:** The results of a comparison of (a) men and (b) women for multi-classification and binary classification using MFCC and Mel-spectrogram.

(a) Men								
Feature Used	Classification Method	Accuracy	Precision	Recall	F1-Score	FDR	FNR	Number of Data
Mfcc	Multi-classification for all group	0.882	0.887	0.882	0.880	0.113	0.118	936
	Binary classification of moderate and mild dementia	0.940	0.943	0.940	0.941	0.057	0.060	500
	Binary classification of moderate dementia and MCI group	0.833	0.694	0.833	0.758	0.306	0.167	544
	Binary classification of mild dementia and MCI group	0.781	0.819	0.781	0.775	0.181	0.220	828
Mel-spectrogram	Multi-classification for all group	0.936	0.937	0.936	0.936	0.063	0.065	936
	Binary classification of moderate and mild dementia	0.980	0.981	0.980	0.980	0.020	0.020	500
	Binary classification of moderate dementia and MCI group	0.963	0.965	0.963	0.961	0.036	0.037	544
	Binary classification of mild dementia and MCI group	0.927	0.928	0.927	0.927	0.072	0.073	828
**(b) Women**								
**Feature used**	**Classification Method**	**Accuracy**	** Precision **	** Recall **	** F1-Score **	**FDR**	** FNR **	** Number of Data **
Mfcc	Multi-classification for all group	0.725	0.766	0.725	0.729	0.235	0.275	3053
	Binary classification of moderate and mild dementia	0.635	0.403	0.635	0.493	0.597	0.365	2083
	Binary classification of moderate dementia and MCI group	0.825	0.827	0.825	0.823	0.173	0.175	2294
	Binary classification of mild dementia and MCI group	0.843	0.845	0.843	0.843	0.155	0.157	1729
Mel-spectrogram	Multi-classification for all group	0.905	0.915	0.905	0.906	0.086	0.095	3053
	Binary classification of moderate and mild dementia	0.962	0.962	0.962	0.962	0.039	0.039	2083
	Binary classification of moderate dementia and MCI group	0.935	0.935	0.935	0.934	0.065	0.066	2294
	Binary classification of mild dementia and MCI group	0.959	0.959	0.959	0.959	0.041	0.041	1729

## Data Availability

The audio data supporting the conclusions of this article include information about the patient’s address, life history, and spouse. Due to the confidentiality of the participants, the release of the raw audio data is limited; however, it may be shared with researchers with the consent of the hospital, patients, and all authors.
